# Therapeutic Management and Outcomes of Hepatoblastoma in a Pediatric Patient with Mosaic Edwards Syndrome

**DOI:** 10.3390/genes15040463

**Published:** 2024-04-07

**Authors:** Patrycja Sosnowska-Sienkiewicz, Alicja Kamińska, Iwona Anderko, Gabriela Telman-Kołodziejczyk, Przemysław Mańkowski, Danuta Januszkiewicz-Lewandowska

**Affiliations:** 1 Department of Pediatric Surgery, Traumatology and Urology, Poznan University of Medical Sciences, 60-572 Poznan, Poland; iwona.anderko@onet.pl (I.A.); mankowskip@ump.edu.pl (P.M.); 2 University Research Hospital in Poznan, Poznan University of Medical Sciences, 60-355 Poznan, Poland; alicjaaleksandrakaminska@gmail.com; 3 Department of Pediatric Cardiology, Nephrology and Hypertension, Poznan University of Medical Sciences, 61-701 Poznan, Poland; gabriela.telman@gmail.com; 4 Department of Pediatric Oncology, Hematology and Transplantology, Poznan University of Medical Sciences, 60-572 Poznan, Poland; danuta.januszkiewicz@ump.edu.pl

**Keywords:** child, hepatoblastoma, liver, trisomy 18, treatment

## Abstract

The mosaic form of Edwards syndrome affects 5% of all children with Edwards syndrome. The clinical phenotype is highly variable, ranging from the full spectrum of trisomy 18 to the normal phenotype. The purpose of this publication was to present the therapeutic process in an 18-month-old girl with the mosaic form of Edwards syndrome and hepatoblastoma, against the background of other cases of simultaneous occurrence of this syndrome and hepatoblastoma described so far. It appears that this particular group of patients with hepatoblastoma and Edwards syndrome can have good outcomes, provided they do not have life-threatening cardiac or other severe defects. Due to the prematurity of our patient and the defects associated with Edwards syndrome, the child required constant multidisciplinary care, but Edwards syndrome itself was not a reason to discontinue therapy for a malignant neoplasm of the liver. Regular abdominal ultrasound examination, along with AFP testing, may be helpful in the early detection of liver tumors in children with Edwards syndrome

## 1. Introduction

Edwards syndrome (also known as trisomy 18) is a genetic disorder in which a child inherits an extra copy of chromosome 18 [1] and is the second most frequent chromosomal anomaly after Down syndrome. This extra copy may be present in some or all of the child’s cells and can lead to a severe clinical condition in the child. There are three different forms of Edwards syndrome. In the full form of Edwards syndrome, the child has inherited a complete extra copy of chromosome 18, and all cells contain an extra chromosome 18. Approximately 94% of children born with Edwards syndrome have the full form of the syndrome. Mosaic Edwards syndrome (5% of all cases of Edwards syndrome) means that the child inherits a complete extra copy of chromosome 18, but only some of the baby’s cells contain the copy. This results in a mixture of normal and trisomy cells, causing highly variable clinical outcomes. In the partial form of Edwards syndrome, only a segment of the extra chromosome is present in the cells, an exceedingly rare scenario [1,2,3].

Typical features of Edwards syndrome include congenital heart defects, developmental delays, feeding difficulties, small head size (microcephaly), and distinctive facial features such as low-set malformed ears, micrognathia, upturned nose, palpebral fissures, hypertelorism, and ptosis. Other deviations include a short breast bone, choroid plexus cysts, clenched fists with overlapping fingers, underdeveloped thumbs and/or nails, absent radius, webbing of the second and third toes, clubfoot or rocker bottom feet, and in males, undescended testicles and hypospadias. Associated congenital anomalies include midline defects (cleft lip/palate), renal anomalies, congenital heart disease, and inguinal and umbilical hernia. Among affected children, 75–95% of die in the first year of life, most often as a result of complex congenital heart defects and structural brain defects [3,4,5,6].

The occurrence of neoplasms in people with Edwards syndrome is rare, and the overall risk varies from person to person. It is estimated that there is a 1% risk of developing Wilms tumor in patients with trisomy 18, and the risk of hepatoblastoma is even higher [7]. Nevertheless, this syndrome has a higher risk of tumors such as liver neoplasms, primarily hepatoblastoma; kidney neoplasms, nephroblastoma; neuroblastoma; and hematological malignancies such as Hodgkin’s disease and acute myeloid leukemia. The incidence of neoplasms does not appear to correlate with body weight, tissue growth, or chromosome 18 cancer gene mapping [7].

The mosaic form of Edwards syndrome can be diagnosed by genetic testing, usually by amniocentesis or chorionic villus sampling (CVS) during pregnancy or by blood tests after birth. The management and treatment of mosaic Edwards syndrome focuses on addressing the specific medical and developmental needs of the individual, which may include surgery to correct heart defects, physical and occupational therapy to promote development, and ongoing medical care to manage related health problems. The prognosis for individuals with mosaic Edwards syndrome varies depending on the severity of symptoms and associated complications. Recent clinical reports of successful treatment of malignancies in children with Edwards syndrome often raise ethical questions [6,7]. Trisomy 18, due to a number of accompanying defects, is the cause of high mortality in children before the age of 1, and only some parents choose to treat, for example, heart defects. Hence, additional cancer treatment may be debatable.

Hepatoblastoma is the most common liver cancer in children, predominantly affecting patients under the age of three. The incidence of hepatoblastoma is relatively low, with approximately 1.5 cases per million children, although the incidence has been increasing slightly over the past few decades. Treatment for hepatoblastoma typically involves a combination of surgery and chemotherapy. The specific chemotherapy regimen often follows protocols such as SIOPEL or COG. The 5-year survival rate for children with hepatoblastoma is approximately 70–80% when the disease is localized. Survival rates are lower for metastatic disease but have been improving. Prognosis is influenced by several factors, including the presence of metastases at diagnosis, the tumor’s response to chemotherapy, and the feasibility of complete surgical resection. Long-term follow-up is crucial for survivors of hepatoblastoma, as late effects of treatment can include hearing loss, cardiac dysfunction, and secondary malignancies. Regular monitoring for recurrence is also important, particularly in the first two years post-treatment, when the risk is highest. Research into the pathophysiology of hepatoblastoma continues, with a focus on genetic and molecular factors that may guide future targeted therapies. This area of research is particularly important for cases associated with genetic syndromes, such as Edwards syndrome, where the interplay between the genetic disorder and cancer development is complex and not yet fully understood [6,7].

Literature data on the occurrence of hepatoblastoma in Edwards syndrome are scarce, and even casuistic in mosaic trisomy 18. So, this manuscript aims to detail the diagnostic journey and therapeutic management of a pediatric patient with mosaic Edwards syndrome and hepatoblastoma, illuminating the multifaceted nature of care. We also wanted to emphasize that Edwards syndrome alone is not a reason to stop treating malignant liver cancer, which can be successfully cured in those patients.

## 2. Case Report

A Caucasian girl, born from a mother with a history of two pregnancies and two deliveries (G2, P2), was delivered by cesarean section at 27 weeks gestation due to sudden and heavy vaginal bleeding, worsening cardiotocography (KTG) values, and the mother’s deteriorating condition. During pregnancy, due to abnormal PAPPA (1:24—risk of Edwards syndrome) and microsomy, the patient was referred for karyotype testing and amniocentesis. Amniocentesis showed no abnormalities, and the karyotype was normal (46, XX,16qh+—a polymorphic variant with no clinical significance). After delivery, the girl scored 6 points on the Apgar scale with a birth weight of 645 g. Due to observed dysmorphisms such as microcephaly, micrognathia, and patent ductus arteriosus (PDA), a chromosomal analysis of peripheral blood cells was conducted. The results showed a 47, XX,+18 karyotype, consistent with Edwards syndrome. The prematurity was associated with bronchodysplasia. The family history was unremarkable.

**Figure 1:**
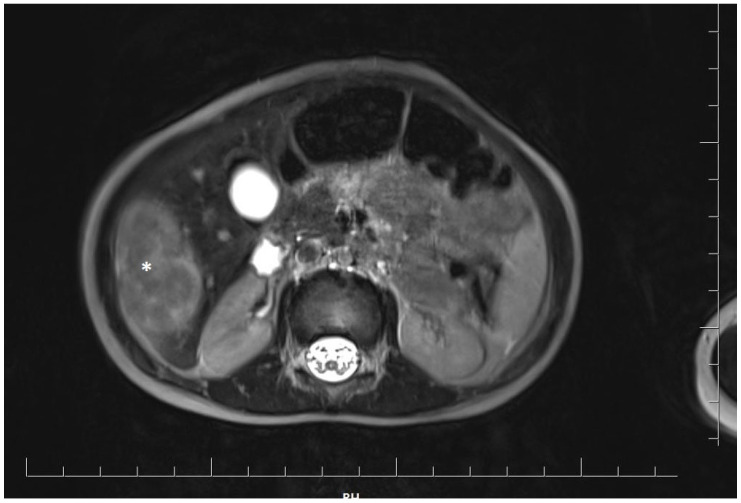
Magnetic resonance imaging of the abdominal cavity showing well-defined, polycyclic, heterogeneous lesions in the right hepatic lobe. The tumor is marked with an asterisk.

At 18 months of age, the girl was admitted to the oncology department for a detailed diagnostic evaluation of three incidentally detected nodular lesions in the right lobe of the liver, measuring 25 mm, 28 mm, and 15 mm in diameter. The tumors were initially identified during a routine follow-up abdominal ultrasound. Magnetic resonance imaging (MRI) of the abdominal cavity was performed and showed well-defined, polycyclic, heterogeneous lesions in segments V and VI of the right hepatic lobe. The lesions were hyperintense compared to the parenchyma and measured approximately 4.8 × 4.1 × 2.5 cm (cc × ds. × ap) (Figure 1).

In addition, an elevated α-fetoprotein (AFP) level of 182.6 24 ng/mL (normal range <8.0 ng/mL) was noted. Other laboratory tests showed HGB: 11.1 mg/dL, PLT: 363 × 109/L, and WBC: 4.23 × 109/L. OB, AST, ALT, Bilirubin, Creatinine, Urea, GGTP, Ferritin, and NSE were within normal range. Given the elevated AFP levels and imaging findings, a liver biopsy was recommended to rule out hepatoblastoma. A percutaneous fine-needle biopsy of the liver tumor under general anesthesia and ultrasound guidance confirmed the diagnosis of a highly differentiated fetal-type hepatoblastoma. Immunohistochemistry showed the following: INI (−), Glypican-3 (+/−), CK7 (−), CK18 (+), hepatocyte (+), CD34 (+), β-catenin (−/+/++), Vimentin (−). The CT scan of the chest revealed three perivascular nodules, two with a diameter of 1 mm and one with a diameter of 2 mm, at the border of lobes LS6 and LS10.

**Figure 2:**
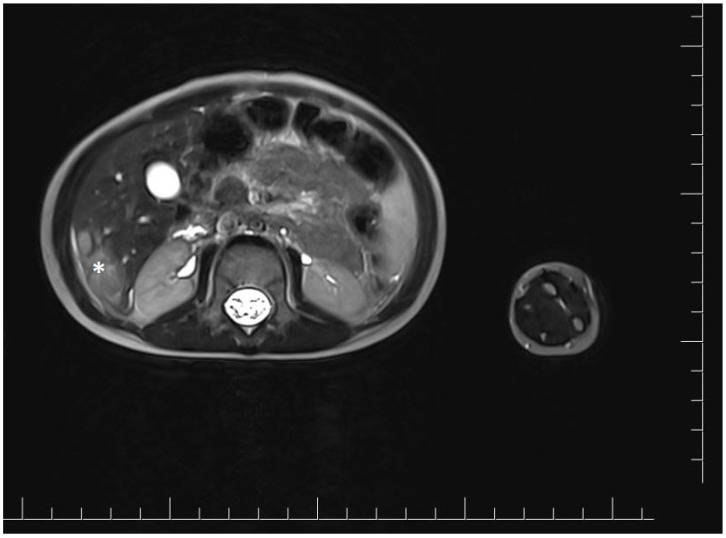
Magnetic resonance imaging of the abdominal cavity showing lesions in the right hepatic lobe after two cycles of cisplatin chemotherapy. The tumor is marked with an asterisk.

Vascular port implantation was performed. Cisplatin chemotherapy was started according to the protocol for hepatoblastoma. After two months, abdominal magnetic resonance imaging was performed to evaluate the treatment response after two cycles of chemotherapy. It showed a reduction of the lesion in the right lobe, in segments V and VI, to approximately 2.7 × 1.4 × 2.8 cm (cc × ds × ap) (Figure 2). Chest X-ray did not show any suspicious lesions.

In addition, a significant decrease in AFP levels was achieved after chemotherapy, measuring 18.24 ng/mL (normal range <8.0 ng/mL), and the patient was then qualified for surgery to remove the liver tumor. During laparotomy, a palpable tumor was identified in the right lobe of the liver. The gallbladder was removed, along with an enlarged palpable lymph node. Intraoperative ultrasound was used to identify the optimal site for radical resection. Temporary occlusion of the right hepatic lobe vessels was performed. Doppler ultrasound was used to delineate the course of the hepatic veins. Segmental resection was performed with a water jet knife within the boundaries of healthy tissue. Postoperatively, the patient remained in good clinical condition.

Histopathologic analysis confirmed the diagnosis of hepatoblastoma—fetal type after initial chemotherapy, indicating partial regression of the tissue. The resection was considered complete, with a minimum margin of 2 mm. The removed lymph node was free of cancer cells.

**Figure 3:**
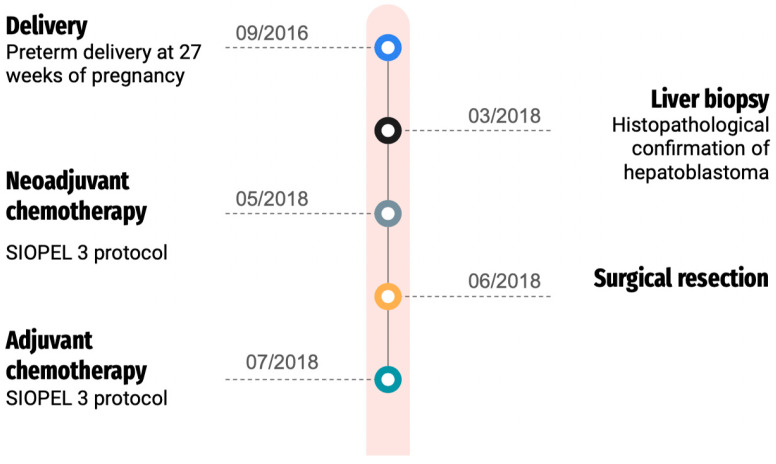
Patient’s treatment timeline.

After surgery, the girl underwent additional rounds of chemotherapy, including cisplatin, according to the SIOPEL 3 treatment protocol. Subsequent follow-ups included abdominal MRI, chest CT, and abdominal ultrasound. The patient, now 7 years old, has successfully remained in complete remission for more than 5 years post-treatment, and ongoing outpatient surveillance has shown no evidence of hepatoblastoma recurrence. Our patient presented typical features of Edwards syndrome microcephaly, micrognathia, and patent ductus arteriosus (PDA), developmental delays, low-set malformed ears, and hypertelorism. In addition, the child has clenched fists with overlapping fingers. She remains also under strict otolaryngology, gastroenterology, ophthalmology, and pulmonology care. Her hearing remains intact. The patient has been diagnosed with retinopathy of prematurity, in the left eye = 7D, 60% amblyopia. Figure 3 presents the patient’s treatment timeline.

## 3. Discussion

Edwards syndrome is a rare disorder with a reported incidence of 1/3000 to 1/7000 births [8]. Survival varies widely, with an average lifespan of 1–3 months for those severely affected [9]. Approximately 95% die in utero, only 50% of live-born infants survive to 2 months, and only 5–10% survive to their first birthday [10,11,12]. A small group of patients have survived to the age of 15–19 years [1,5]. Patients with mosaicism have lived longer. Our female patient with the mosaic form of Edwards syndrome was born in 2016 and is currently 7 years old.

The incidence of Edwards syndrome may be influenced by factors such as the mother’s advanced age, environmental factors, and low socioeconomic level [13,14]. In this case, both advanced maternal age and low socioeconomic status were noted.

The prenatal diagnosis of trisomy 18 by means of amniocentesis in the second half of the pregnancy is now a very common event [13]. Genetic diagnosis for our patient was conducted postnatally, prompted by the observation of dysmorphic features.

Many structural defects have been reported in association with trisomy 18. In 97% of cases with trisomy 18, structural defects are observed in at least three organs [1,3,4,5,6]. Our patient exhibited several of these defects, including Patent Ductus Arteriosus (PDA), developmental delay, microcephaly, and micrognathia.

Satgé et al. suggest that female sex may offer a survival advantage in trisomy 18, evidenced by a higher incidence of females in reported cases of Edwards syndrome and hepatoblastoma [7]. This observation is supported by our patient being female. However, the phenomenon of female predominance in children with T18 and HB does not exclude any possibility of an alternative molecular pathway that might promote tumorigenesis in these cases. Interestingly, cytogenetic analyses of hepatoblastomas not pre-selected for any genetic condition have shown that trisomy 18, or at least the gain of chromosome 18, is a rarity [15]. This brings into question whether trisomy 18 independently contributes to the development of hepatoblastoma through the numerical chromosomal aberration itself. Trisomy 18 has been found mainly in hematopoietic cancers, but also in breast cancer and pilomatricoma. Since children with Edwards syndrome have a short life expectancy; it is not possible to assess whether constitutional trisomy 18 increases the risk of adult cancers. However, the role of constitutional trisomy 13, 18, and 21 remains to be understood to explain the very different tumor distribution.

Further complicating the discussion, Pereira et al. documented a case of hepatoblastoma in a girl with mosaic T18, where the tumor cells did not contain an extra copy of chromosome 18 [3]. This suggests that while the presence of trisomy 18 may not directly lead to tumorigenesis in hepatoblastoma, the underlying genetic landscape of individuals with Edwards syndrome, particularly those with a mosaic form, might influence cancer development through mechanisms not solely dependent on the presence of an additional chromosome 18. Consistent with these findings, our case emphasizes the need for further investigation into the complex interplay between genetic aberrations and cancer susceptibility in Edwards syndrome, particularly given the potential for alternative pathways of tumorigenesis that do not rely on chromosomal abnormalities traditionally associated with the condition.

Due to the high early mortality rate among children with Edwards syndrome, our understanding of their cancer risk profile remains limited. Nevertheless, there has been a reported increase in cases of malignant tumors, particularly affecting the liver and kidneys [7].

In a patient, diagnosed at 1.5 years with hepatoblastoma, the tumor was incidentally discovered during a routine ultrasound. To date, approximately 50 cases of hepatoblastoma in Edwards syndrome have been reported [7,16]. Because live-born infants with Edwards syndrome may be at risk for developing hepatoblastoma, Farmakis et al. suggested performing abdominal ultrasound and serial AFP measurements every 3 months at least until 4 years of age, considering the lack of validated, age-related AFP levels in children with trisomy 18 [16]. This protocol has been proven as an effective one in the case of our patient. In our patient, an initial liver biopsy was performed. Histopathological examination confirmed hepatoblastoma. Two cycles of cisplatin chemotherapy were introduced. After obtaining a positive response as tumor shrinkage, the child was qualified for surgical resection of the lesion. A segmental liver resection with a margin of healthy tissue was performed. Similar management, in accordance with the Childhood Liver Tumor Strategy Group (SIOPEL) guidelines, has been described in other children with Edwards syndrome and a diagnosis of hepatoblastoma [2,3,8].

Ahmad et al. described that surgery was successfully attempted in all seven living patients with Edwards syndrome and hepatoblastoma. Patients who died had severe concomitant cardiac or other defects. These defects were determined to be the cause of death [2]. Our patient was diagnosed with PDA, which required only pharmacotherapy, without surgical treatment. Our patient is now 7 years old and has been in complete remission for more than 5 years since the end of treatment. She is under constant outpatient observation. We are optimistic that she has been cured of her hepatoblastoma.

A review of the cases of trisomy 18 and congenital hepatoblastoma that were available in the literature is summarized in Table 1 [2,3,8,17,18,19,20,21,22,23,24,25,26,27,28,29,30].

## 4. Conclusions

It appears that this particular group of patients with hepatoblastoma and Edwards syndrome can have good outcomes, provided they do not have life-threatening cardiac or other severe defects.

Due to the prematurity of our patient and the defects associated with Edwards syndrome, the child requires constant multidisciplinary care, but Edwards syndrome itself is not a reason to discontinue therapy for a malignant neoplasm of the liver.

Regular abdominal ultrasound examination, along with regular testing of AFP levels, may be helpful in the early detection of liver tumors in children with Edwards syndrome.

## Figures and Tables

**Table 1:** Review of cases with trisomy 18 and congenital hepatoblastoma.

Trisomy 18	
Total	*n* = 18
Mosaic	*n* = 4
**Gender**	
Female	*n* = 17
Male	*n* = 5
**Age at the moment of hepatoblastoma diagnosis**	
0–6 months	*n* = 5
7–12 months	*n* = 9
1–2 years	*n* = 5
2–4 years	*n* = 3
above 4 years	*n* = 0
**Treatment**	
Surgery	*n* = 5
Chemotherapy	*n* = 0
Surgery + Chemotherapy	*n* =12
Surgery + Chem + Liver transplant	*n* = 1
No treatment	*n* = 4
**Disease status**	
remission	*n* = 16
progression	*n* = 6
**Death**	
due to cancer	*n* = 2
due to organ failure	*n* = 3
related to 18 trisomy	*n* = 1
no reported death	*n* = 16
**Age of delivery**	
preterm	*n* = 9
at term	*n* = 11
n/a	*n* = 2

## Data Availability

Data available on request due to restrictions.
